# Case Report: Resectability-guided management of an indeterminate periduodenal spindle cell tumor initially suspected to be a gastrointestinal stromal tumor

**DOI:** 10.3389/fonc.2026.1916609

**Published:** 2026-08-07

**Authors:** Jie Li, Guangyi Li

**Affiliations:** The Affiliated Hospital of Yanbian University (Yanbian Hospital), Yanbian, China

**Keywords:** case report, duodenum, gastrointestinal stromal tumor, pancreaticoduodenectomy, spindle cell tumor

## Abstract

Duodenal mesenchymal spindle cell tumors are uncommon and may overlap radiologically with gastrointestinal stromal tumors (GISTs) and retroperitoneal lesions. We report a 55-year-old woman with abdominal discomfort, nausea, and vomiting. Contrast-enhanced computed tomography demonstrated an approximately 6.0 × 3.8 cm mass posterior to the pancreatic head that was interpreted as a probable duodenal stromal tumor. Gastroscopic biopsy was non-representative. Given the symptoms, tumor size, uncertain origin, and vascular proximity, surgical exploration was undertaken. The tumor was firm and densely adherent to the inferior vena cava, and pancreaticoduodenectomy was performed. Supplementary pathological review showed a submucosal spindle cell tumor with 2 mitoses per 50 high-power fields, hemorrhage and inflammatory exudates, focal infiltrative growth, and limited invasion of adjacent pancreatic tissue. DOG-1, CD117, desmin, and CD34 were negative; SMA was positive, and β-catenin showed only weak cytoplasmic staining without nuclear accumulation. The expanded panel remained insufficient for definitive lineage assignment, and the lesion was designated an unclassified duodenal mesenchymal spindle cell tumor with SMA expression. Four abdominal computed tomography examinations and one magnetic resonance imaging examination showed no definite recurrence or metastasis through May 28, 2025. This case illustrates the limitations of superficial biopsy and shows that management of an anatomically complex periduodenal tumor may need to integrate diagnostic uncertainty, resectability, vascular proximity, and operative safety.

## Introduction

Mesenchymal tumors of the duodenum are uncommon, and spindle cell tumors that cannot be readily classified as conventional GIST are particularly rare. Their clinical manifestations are nonspecific, and their imaging features may overlap with those of GISTs, peripancreatic masses, and retroperitoneal lesions (1, 2). When these lesions arise from the deeper layers of the duodenal wall or grow predominantly outside the lumen, routine endoscopic biopsy may be limited by insufficient sampling depth and fail to obtain representative tissue (2–6). Here, we report a duodenal spindle cell tumor that was considered likely to represent GIST preoperatively but remained unclassified after resection on the basis of the available pathological material. We focus on the discordance between cross-sectional imaging and superficial biopsy, the limits of pathological subclassification, and the anatomical and operative factors that informed surgical management. Because the lesion abutted major vascular structures and ultimately required pancreaticoduodenectomy, the case is relevant to surgical oncology practice in the management of indeterminate periduodenal tumors. The manuscript was prepared in accordance with the CARE reporting guideline (16).

## Case description

A 55-year-old woman developed abdominal discomfort 1 day before admission. Non-contrast upper abdominal computed tomography performed at an outside hospital demonstrated a mass-like lesion anterior to the right kidney that was indistinct from the pancreatic head and duodenum. She was admitted the next day for further evaluation and treatment. On admission, she reported nausea, vomiting, and abdominal distension, and her abdominal discomfort was partially relieved after vomiting. She had no fever and no recent significant weight loss. Her medical history was unremarkable for hypertension, diabetes mellitus, hepatitis, tuberculosis, previous surgery, or trauma, and she reported no known food or drug allergies. Physical examination revealed a soft abdomen without tenderness, rebound tenderness, guarding, or a palpable abdominal mass. No additional clinically relevant abnormalities were documented in the retrievable admission examination.

Timeline

A detailed timeline of the diagnostic, therapeutic, and follow-up course is provided in **Supplementary Figure 1**.

## Diagnostic assessment

Laboratory testing on admission showed a white blood cell count of 15.08 × 10^9/L, a neutrophil percentage of 80.2%, and a high-sensitivity C-reactive protein level of 13.77 mg/L, suggesting an inflammatory response. Alanine aminotransferase was mildly elevated at 76 U/L. CEA, CA19-9, CA72-4, and CA125 were all within normal limits.

**Figure 1:**
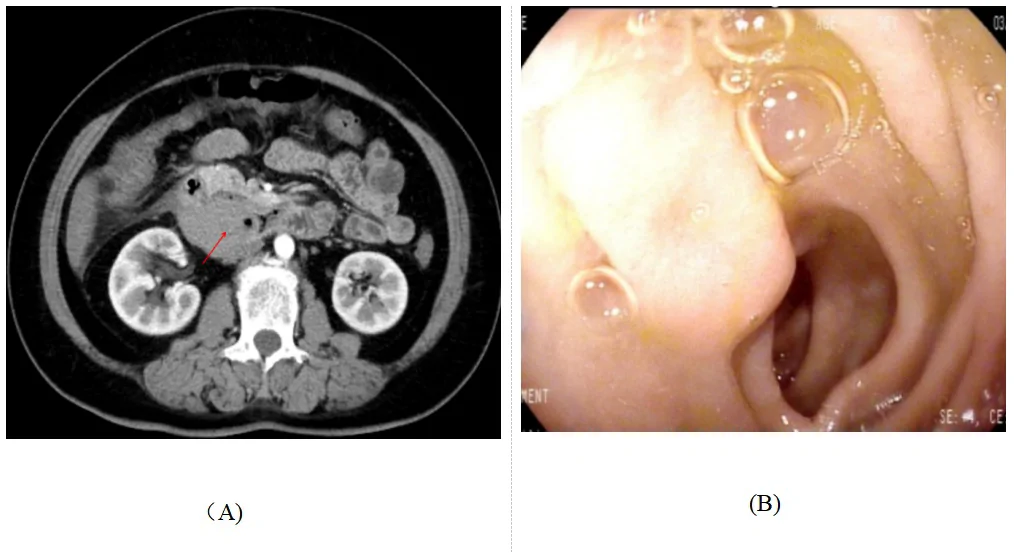
Preoperative imaging and endoscopic findings. **(A)** Contrast-enhanced computed tomography demonstrated an approximately 6.0 × 3.8 cm relatively well-defined, predominantly solid mass posterior to the pancreatic head. The arterial, portal venous, and delayed phases all showed relatively homogeneous mild enhancement. No progressive enhancement or rapid washout pattern was observed, and there was no apparent heterogeneous enhancement, necrosis, hemorrhage, cystic degeneration, or calcification. The mass occupied the right periduodenal/retroperitoneal space and abutted the inferior vena cava, but its precise organ of origin could not be established. The original radiology report described the lesion as closely related to the duodenum and favored a stromal tumor. **(B)** Gastroscopy showed mucosal changes in the duodenal bulb. Routine endoscopic mucosal biopsy revealed only heterotopic fundic glands with chronic inflammation and papillary epithelial hyperplasia, which did not account for the deep-seated lesion.

Contrast-enhanced whole-abdomen computed tomography obtained during hospitalization demonstrated an approximately 6.0 × 3.8 cm relatively well-defined, predominantly solid soft-tissue mass posterior to the pancreatic head. The arterial, portal venous, and delayed phases all showed relatively homogeneous mild enhancement. No progressive enhancement or rapid washout pattern was observed, and there was no apparent heterogeneous enhancement, macroscopic hemorrhagic component, cystic degeneration, or calcification on visual assessment. The mass occupied the right periduodenal/retroperitoneal space, abutted the inferior vena cava, and showed no convincing pancreatic parenchymal origin. A predominantly extraluminal growth pattern was suspected; however, computed tomography did not establish definite continuity with the duodenal muscular wall or a clear origin from the inferior vena cava or another retroperitoneal structure. The contemporaneous radiology report described the lesion as closely related to the duodenum and favored a stromal tumor. Taken together, these findings made a duodenal subepithelial mesenchymal tumor, particularly GIST, a reasonable leading preoperative consideration, while an inferior vena cava-origin or other retroperitoneal mesenchymal tumor remained in the differential diagnosis (**Figure 1**).

Gastroscopy performed during the same admission suggested possible Barrett’s esophagus, chronic non-atrophic gastritis, and mucosal changes in the duodenal bulb (**Figure 1**). Pathological examination of the endoscopic biopsy showed only heterotopic fundic glands with chronic inflammation and papillary epithelial hyperplasia in the posterior wall of the duodenal bulb. These findings did not explain the deep-seated lesion identified on cross-sectional imaging and were therefore considered non-representative of the underlying mass. No preoperative magnetic resonance imaging study, EUS report, or EUS-guided tissue acquisition result was identified in the retrievable record.

## Therapeutic intervention

Given the patient’s persistent gastrointestinal symptoms, the approximately 6 cm tumor, its indeterminate origin, and its close relationship to the duodenum, pancreatic head, and major vascular structures, a mesenchymal neoplasm with malignant potential could not be excluded. Because the feasibility of safe local resection was uncertain, surgical exploration was undertaken.

Laparoscopic pancreaticoduodenectomy was planned. Intraoperatively, the tumor was firm and densely adherent to the inferior vena cava. Continued laparoscopic dissection carried a substantial risk of vascular injury, and the procedure was converted to open pancreaticoduodenectomy. Concomitant cholecystectomy was performed for documented cholelithiasis. The operative duration was 340 min, and the patient received 1.5 units of packed red blood cells and 200 mL of plasma intraoperatively.

**Figure 2:**
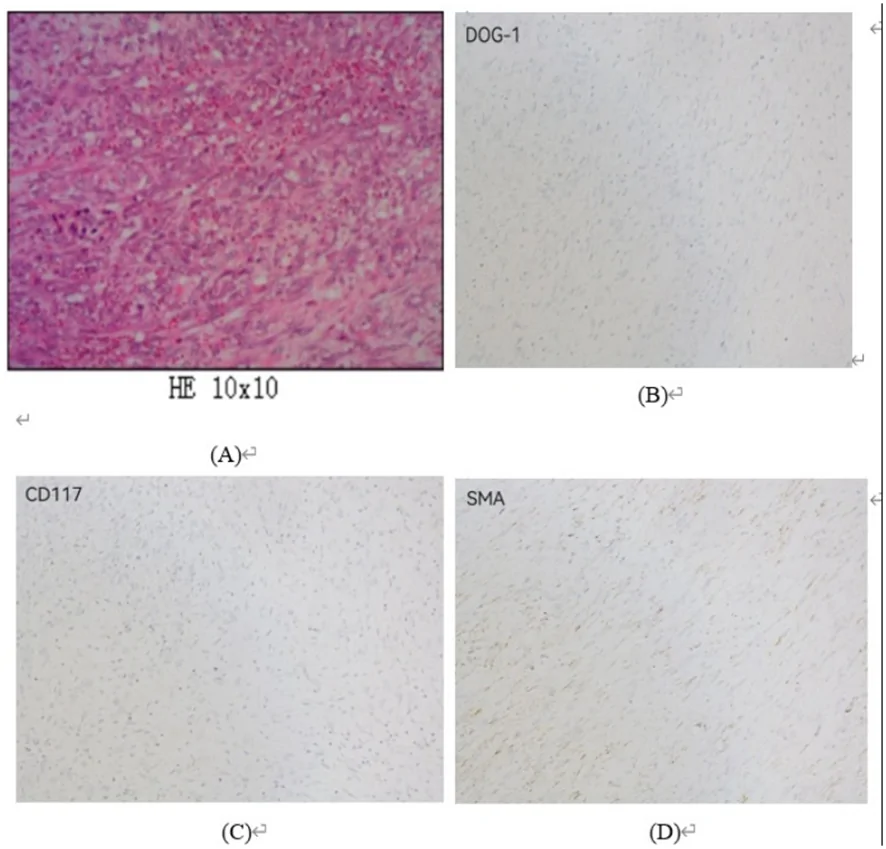
Histopathological and immunohistochemical findings. **(A)** Hematoxylin and eosin staining showed a spindle cell tumor with focal fascicular or interlacing architecture, moderate atypia, and focal myxoid change. **(B)** DOG-1 staining was negative. **(C)** CD117 staining was negative. **(D)** SMA staining was positive; however, SMA expression alone was insufficient to establish smooth-muscle lineage. Taken together, the available findings made conventional gastrointestinal stromal tumor less likely but did not exclude an uncommon immunonegative gastrointestinal stromal tumor or permit definitive subclassification.

Postoperative pathological examination demonstrated a mesenchymal spindle cell tumor arising in the duodenum and measuring 6 × 6 × 4 cm grossly. Tumor cells showed moderate cytological atypia and focal myxoid change. Preoperative CT showed a mass closely related to the duodenum but did not establish its wall-layer origin; postoperative pathological review, rather than CT or EUS, localized the tumor to the duodenal submucosa and documented 2 mitoses per 50 high-power fields. Hemorrhage and inflammatory exudates were present, whereas unequivocal tumor-cell necrosis was not described. The tumor showed focal infiltrative growth with limited invasion of adjacent pancreatic tissue. Immunohistochemistry showed negativity for cytokeratin, DOG-1, CD117, S-100, Bcl-2, desmin, and CD34; positivity for vimentin and SMA; focal EMA expression; and weak cytoplasmic β-catenin staining without nuclear accumulation. The Ki-67 labeling index was focally increased to approximately 20%. DOG-1, CD117, and CD34 negativity made conventional GIST less likely but did not exclude an uncommon immunonegative GIST. Desmin negativity further weakened support for smooth-muscle differentiation, although h-caldesmon was not performed. S-100 negativity did not support neural differentiation, and the absence of nuclear β-catenin expression did not support desmoid-type fibromatosis (**Figure 2**). H-caldesmon, SOX10, ALK, STAT6, and SDHB immunohistochemistry, together with KIT and PDGFRA molecular testing, were not available within our institutional pathology laboratory and were not included in the original work-up. Because this was a retrospective case, external testing could not be completed during the current revision. These unperformed studies were not interpreted as negative findings. Accordingly, the lesion was conservatively designated an unclassified duodenal mesenchymal spindle cell tumor with SMA expression. The focal pancreatic invasion supported locally infiltrative behavior but did not permit classification as leiomyosarcoma or another defined sarcoma or application of a validated tumor-specific prognostic model. No tumor was identified at the proximal or distal intestinal resection margins, gastric margin, or pancreatic margin. Twelve lymph nodes showed reactive changes without metastasis. Chronic cholecystitis was also present.

## Follow-up and outcomes

**Figure 3:**
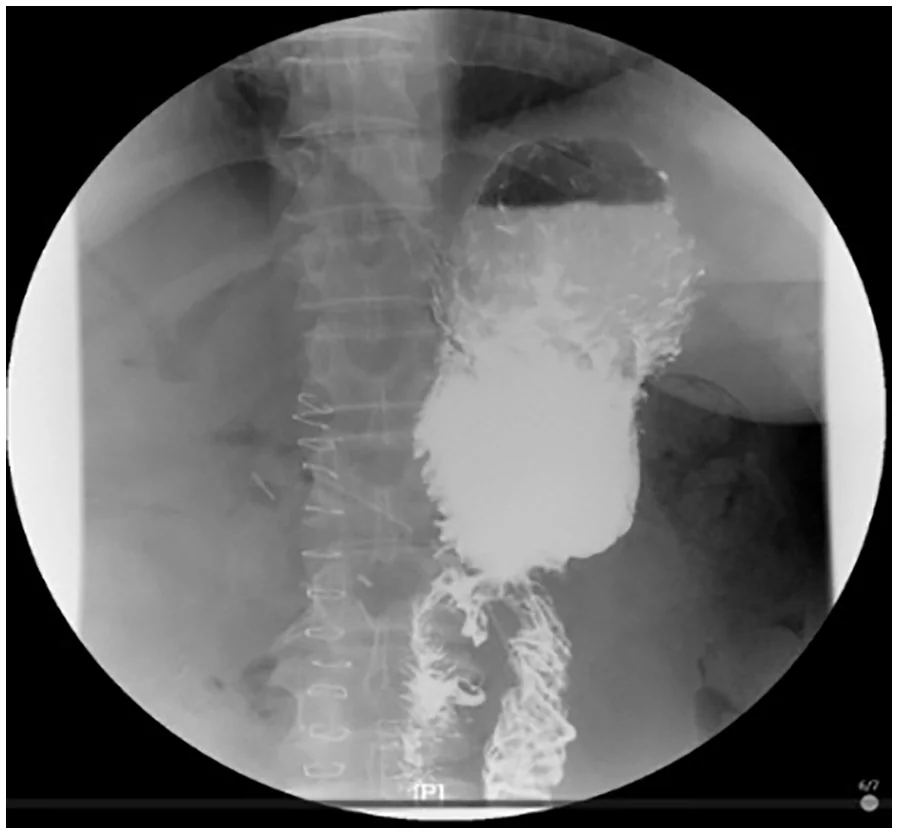
Postoperative upper gastrointestinal contrast study. An upper gastrointestinal contrast study performed on postoperative day 13 showed no obvious anastomotic obstruction or contrast leakage, indicating a patent gastrointestinal reconstruction.

The postoperative course was generally stable. Early after surgery, the patient experienced transient fever, nausea, and variable drain output, which gradually resolved with antimicrobial therapy, fluid replacement, nutritional support, and symptomatic management. On postoperative day 13, an upper gastrointestinal contrast study showed no obvious anastomotic obstruction or contrast leakage, indicating a patent gastrointestinal reconstruction (**Figure 3**). The patient was discharged in good general condition on postoperative day 16.

She subsequently underwent regular imaging surveillance. As of May 28, 2025, four abdominal computed tomography examinations and one abdominal magnetic resonance imaging examination had shown no definite local recurrence or distant metastasis.

## Discussion

This case illustrates the limits of preoperative characterization of a deep spindle cell tumor adjacent to the duodenum within the documented work-up. On computed tomography, the lesion was a predominantly solid, relatively well-defined mass posterior to the pancreatic head, occupying the right periduodenal/retroperitoneal space and abutting the inferior vena cava. It showed mild and relatively homogeneous enhancement across the available postcontrast series, without an obvious nonenhancing necrotic component or coarse calcification on the selected images. Although a predominantly extraluminal growth pattern was suspected, computed tomography could not establish a definite duodenal-wall, inferior vena cava, or other retroperitoneal origin. Importantly, the original radiology impression described the mass as closely related to the duodenum and favored a stromal tumor, which explains why duodenal GIST became the leading working diagnosis rather than a retrospective assumption by the authors. An inferior vena cava-origin or other retroperitoneal mesenchymal tumor nevertheless remained a reasonable alternative. Supplementary pathological review subsequently localized the tumor to the duodenal submucosa and demonstrated focal infiltrative growth with limited invasion of adjacent pancreatic tissue. Postoperative immunohistochemistry made conventional GIST less likely but neither excluded an uncommon immunonegative GIST nor established an alternative lineage. Comparative data from selected published rare non-GIST duodenal or periduodenal tumors relevant to this differential diagnosis are summarized in **Table 1**.

**Table 1:** Comparison of the present case with selected published rare duodenal or periduodenal spindle cell tumors relevant to the differential diagnosis.

Study	Age/sex	Pathology and location	Size	Preoperative impression	Treatment	Key pathology/immunophenotype	Follow-up/outcome
Present case	55/F	Unclassified duodenal mesenchymal spindle cell tumor with SMA expression; centered in the duodenal submucosa	CT: 6.0 × 3.8 cm; gross: 6 × 6 × 4 cm	Duodenal GIST; endoscopic mucosal biopsy non-representative	Pancreaticoduodenectomy after intraoperative confirmation of dense adhesion to the inferior vena cava	Moderate atypia, focal myxoid change, 2 mitoses/50 HPFs, hemorrhage and inflammatory exudates, focal infiltrative growth with limited pancreatic invasion; DOG-1-, CD117-, desmin-, CD34-, SMA+, S-100-, β-catenin weak cytoplasmic+/nuclear-, focal Ki-67 approximately 20%; lineage unclassified	Four abdominal CT examinations and one abdominal MRI through 2025-05-28; no definite recurrence or metastasis
Banerjee et al., 2015 (9)	19/F	Plexiform angiomyxoid myofibroblastic tumor, first part of the duodenum/posterior wall	13.8 × 8.6 cm	Duodenal GIST on ultrasonography, endoscopy, and contrast-enhanced CT	Distal gastrectomy, proximal duodenectomy, and Billroth II gastrojejunostomy	Plexiform spindle-cell tumor in fibromyxoid stroma; PgR+, SMA-, c-KIT-, DOG1-, S-100-, CD34-	No recurrence or metastasis at 6 months
Li et al., 2025 (11)	30/F	Duodenal schwannoma, anterior wall of the duodenal bulb	1.0 × 1.6 cm	Ectopic pancreas or stromal tumor; CT suggested a benign submucosal lesion	Endoscopic submucosal dissection	Spindle-cell tumor; S-100+, DOG-1-, CD117-, CD34-, SMA-	Complete resection; favorable postoperative recovery; no recurrence reported
Oh et al., 2025 (12)	49/M	Inflammatory myofibroblastic tumor, second portion of the duodenum	NR	Duodenal mesenchymal tumor such as GIST	Duodenal wedge resection	Final diagnosis of IMT; ALK-negative; positive margin noted in follow-up report	Long-term benign course without reported recurrence despite positive margin
MacKenzie et al., 2026 (13)	67/M	Duodenal leiomyoma, second portion of the duodenum adjacent to the pancreatic head	11.9 × 8.7 cm	Large duodenal soft-tissue mass with hemorrhage; differential included GIST and leiomyosarcoma	Surgical resection	Bland smooth muscle cells with low Ki-67 and no mitotic activity; smooth muscle antigen+, desmin+	No postoperative complications; resected successfully
Ganamani et al., 2025 (14)	Three female patients	Retroduodenal/juxtapancreatic schwannomas adjacent to the pancreas and duodenum	Individual sizes varied	Pancreatic or retroperitoneal tumors on contrast-enhanced CT	Complete surgical excision in all 3 cases	Antoni A and Antoni B areas with Verocay bodies; S-100+	Smooth postoperative recovery; no recurrence during 1-year follow-up

ALK, anaplastic lymphoma kinase; CT, computed tomography; GIST, gastrointestinal stromal tumor; IMT, inflammatory myofibroblastic tumor; MRI, magnetic resonance imaging; NR, not reported.

This comparison is intentionally selective rather than systematic and emphasizes published cases most relevant to the anatomical differential diagnosis or clinicopathological interpretation of the present tumor.

The present case also underscores the limitations of routine gastroscopic biopsy for deep lesions. The endoscopic biopsy showed only heterotopic fundic glands, chronic inflammation, and superficial epithelial changes, which could not explain the approximately 6 cm deep-seated mass identified on imaging. This discrepancy was consistent with a deep-wall or predominantly exophytic lesion for which routine mucosal sampling did not obtain representative tumor tissue. When imaging findings and endoscopic pathology are clearly discordant, the presence of a clinically relevant deep tumor should not be dismissed solely on the basis of a superficial biopsy result.

Preoperative EUS might have improved lesion characterization by clarifying the layer of origin, its relationship to the duodenal wall, and the feasibility of tissue acquisition (3–6). However, no preoperative MRI study, EUS report, or EUS-guided tissue acquisition result was identified in the retrievable record, and the record did not indicate whether these modalities were considered, why they were absent from the documented work-up, or whether EUS-guided sampling would have been technically feasible. These issues cannot be reconstructed retrospectively. Given the lesion’s size, posterior periduodenal/retroperitoneal location, and proximity to major vascular structures, improved preoperative characterization would not necessarily have made limited resection technically safe or oncologically adequate (7, 8). The case should therefore be interpreted as illustrating the limitations of the documented preoperative work-up rather than a fully exhausted diagnostic pathway.

A key lesson from this case is that the extent of surgery was informed not by the preoperative imaging impression alone but by anatomical resectability, the feasibility of complete removal, and operative safety. Intraoperative findings demonstrated dense adhesion to the inferior vena cava and a complex relationship with the pancreatic head and duodenum. This adhesion should not be interpreted as histological invasion of the inferior vena cava, which was not established. However, supplementary pathological review identified focal invasion of adjacent pancreatic tissue, confirming locally infiltrative behavior. Under these circumstances, limited resection was not considered safely feasible while ensuring complete tumor removal; pancreaticoduodenectomy was therefore selected. Negative margins and the absence of recurrence during the available follow-up document local disease control but neither establish the comparative superiority of this operation nor resolve the tumor’s lineage.

Supplementary pathological review reduced several uncertainties by establishing a submucosal location, a mitotic count of 2 per 50 high-power fields, focal infiltrative growth, and limited invasion of adjacent pancreatic tissue. Hemorrhage and inflammatory exudates were present, but unequivocal tumor-cell necrosis was not described. The pancreatic invasion supports locally infiltrative behavior and is clinically important; however, it does not by itself establish a specific histological lineage or an entity-specific grade. Under current WHO classification principles, spindle cell tumors require integrated assessment of morphology, lineage-specific immunophenotype, mitotic activity, tumor-cell necrosis, and infiltrative behavior (15). DOG-1, CD117, and CD34 negativity made conventional GIST less likely but did not exclude an uncommon immunonegative GIST in the absence of molecular testing; SDHB immunohistochemistry would also have assisted in evaluating an SDH-deficient GIST. SMA expression was not lineage-specific, and desmin negativity further weakened support for smooth-muscle differentiation in the absence of h-caldesmon. S-100 negativity did not support neural differentiation, although SOX10 was not performed. Weak cytoplasmic β-catenin staining without nuclear accumulation did not support desmoid-type fibromatosis. H-caldesmon, SOX10, ALK, STAT6, and SDHB immunohistochemistry and KIT/PDGFRA molecular testing were not available within our institutional pathology laboratory, were not included in the original work-up, and could not be completed externally during the current revision. Their absence was not interpreted as negative evidence. The focally increased Ki-67 labeling index of approximately 20% indicated proliferative heterogeneity but could not independently establish a specific tumor grade. We therefore retain the conservative designation of an unclassified duodenal mesenchymal spindle cell tumor with SMA expression and report focal pancreatic invasion separately as evidence of locally infiltrative behavior. The available findings do not permit classification as leiomyosarcoma or another defined sarcoma or application of a validated tumor-specific prognostic model.

Published reports of rare non-GIST duodenal tumors show that clinical presentation, imaging appearance, and preoperative characterization are often nonspecific and that definitive classification commonly relies on postoperative morphology and immunohistochemistry (9–14). In the present case, persistent discordance between imaging and superficial biopsy, together with tumor size and proximity to major vascular structures, required operative planning to integrate diagnostic uncertainty with the feasibility of safe complete resection. From a surgical oncology perspective, the case illustrates that management of an indeterminate periduodenal tumor may appropriately be guided by anatomical relationships and operative safety when definitive preoperative histology is unavailable.

A strength of this report is the integration of the contemporaneous radiology impression, operative findings, supplementary morphological review, expanded immunohistochemical panel, and serial CT/MRI follow-up. Additional limitations are its retrospective single-case design, the incomplete record regarding preoperative MRI and EUS, reliance on selected CT images for retrospective review, the unavailability of h-caldesmon, SOX10, ALK, STAT6, and SDHB immunohistochemistry and molecular testing within our institutional laboratory, and the duration of available follow-up. These constraints limit generalizability and preclude definitive lineage assignment and entity-specific prognostic classification.

## Patient perspective

The patient and her family were informed that the preoperative diagnosis remained uncertain and that definitive resection was recommended because of the lesion’s location and its close relationship with surrounding vital structures. During postoperative recovery and follow-up, the patient remained clinically stable and expressed acceptance of the diagnostic and therapeutic process.

## Data Availability

The original contributions presented in the study are included in the article/**Supplementary Material**. Further inquiries can be directed to the corresponding author.

## Supplementary material

Supplementary Figure 1 Timeline of diagnosis, treatment, and follow-up. A periduodenal mass was first detected 1 day before admission on outside non-contrast computed tomography. During the index hospitalization, contrast-enhanced abdominal computed tomography demonstrated an approximately 6.0 × 3.8 cm mass posterior to the pancreatic head; the contemporaneous radiology impression described the lesion as closely related to the duodenum and favored a stromal tumor. Gastroscopy and mucosal biopsy were non-representative, and pancreaticoduodenectomy was subsequently performed. During follow-up, four abdominal computed tomography examinations and one abdominal magnetic resonance imaging examination showed no definite local recurrence or distant metastasis through May 28, 2025.

## References

[1] Barat M Pellat A Dohan A Hoeffel C Coriat R Soyer P . CT and MRI of gastrointestinal stromal tumours: new trends and perspectives. Can Assoc Radiol J. (2023) 75:107–17. doi: 10.1177/08465371231180510 37386745

[2] Kim BJ Kang SJ Nam SY Bang CS Min BH Kim SE . Practice guidelines for the diagnosis and treatment of subepithelial lesion observed in upper gastrointestinal endoscopy. J Gastroenterol Hepatol. (2026) 41:488–501. doi: 10.1111/jgh.70225 41502317 PMC12887643

[3] Nakatani S Okuwaki K Watanabe M Imaizumi H Iwai T Matsumoto T . Stereomicroscopic on-site evaluation in endoscopic ultrasound-guided tissue acquisition of upper gastrointestinal subepithelial lesions. Clin Endosc. (2024) 57:89–95. doi: 10.5946/ce.2022.288 37070203 PMC10834295

[4] Facciorusso A Arvanitakis M Crino SF Fabbri C Fornelli A Leeds J . Endoscopic ultrasound-guided tissue sampling: European Society of Gastrointestinal Endoscopy (ESGE) technical and technology review. Endoscopy. (2025) 57:390–418. doi: 10.1055/a-2524-2596 40015316

[5] Chong CC Pittayanon R Pausawasdi N Bhatia V Okuno N Tang RS . Consensus statements on endoscopic ultrasound-guided tissue acquisition: guidelines from the Asian Endoscopic Ultrasound Group. Dig Endosc. (2024) 36:871–83. doi: 10.1111/den.14768 38433315

[6] Verloop CA Hol L Perk L Dircksmeier-Harinck F Honkoop P Romkens T . Population-based efficacy of tissue acquisition for subepithelial lesions in the upper gastrointestinal tract. J Clin Gastroenterol. (2026). doi: 10.1097/MCG.0000000000002340 41707135

[7] Bourke MJ Lo SK Buerlein RCD Das KK . AGA Clinical Practice Update on nonampullary duodenal lesions: expert review. Gastroenterology. (2025) 168:169–75. doi: 10.1053/j.gastro.2024.10.008 39545885

[8] D'Souza LS Yang D Diehl D . AGA Clinical Practice Update on endoscopic full-thickness resection for the management of gastrointestinal subepithelial lesions: commentary. Gastroenterology. (2024) 166:345–9. doi: 10.1053/j.gastro.2023.11.016 38108671

[9] Banerjee N Gupta S Dash S Ghosh S . Plexiform angiomyxoid myofibroblastic tumour of the duodenum: a rare entity. BMJ Case Rep. (2015) 2015:bcr2015210004. doi: 10.1136/bcr-2015-210004 26216925 PMC4521542

[10] Poizat F de Chaisemartin C Bories E Delpero JR Xerri L Flejou JF . A distinctive epitheliomesenchymal biphasic tumor in the duodenum: the first case of duodenoblastoma? Virchows Arch. (2012) 461:379–83. doi: 10.1007/s00428-012-1307-y 22961103

[11] Li S Tu L Li T Pei X Wang X Shi Y . Case report: rare duodenal schwannoma diagnosis and treatment process report. Front Oncol. (2025) 15:1528653. doi: 10.3389/fonc.2025.1528653 40115021 PMC11922719

[12] Oh MH Seo HI Choi KU Hong SB Park YM Noh BG . Inflammatory myofibroblastic tumor on second portion of duodenum: a case report. Korean J Clin Oncol. (2025) 21:159–63. doi: 10.14216/kjco.25374 41508664 PMC12784145

[13] MacKenzie M MacKenzie B Kwan AY Chen CH Yap WW MacKenzie S . Duodenal leiomyoma: a case report of upper gastrointestinal hemorrhage from a giant duodenal tumor. Int J Surg Case Rep. (2026) 138:1481–6. doi: 10.1097/RC9.0000000000000354 41938421 PMC13045972

[14] Ganamani AC Palanichamy SK Subramaniam S . Retroduodenal and juxtapancreatic schwannomas: clinical and surgical insights from three cases. Cureus. (2025) 17:e85916. doi: 10.7759/cureus.85916 40656403 PMC12256019

[15] WHO Classification of Tumours Editorial Board . Digestive System Tumours. 5th ed. Lyon: International Agency for Research on Cancer (2019).

[16] Gagnier JJ Kienle G Altman DG Moher D Sox H Riley D . The CARE guidelines: consensus-based clinical case reporting guideline development. J Med Case Rep. (2013) 7:223. doi: 10.1186/1752-1947-7-223 24228906 PMC3844611

